# Perspiring Feet

**Published:** 1891-06

**Authors:** R. A. Chudleigh


### PERSPIRING FEET.

Excess of perspiration is one thing; malodorous perspiration is another. We generally veil them under the alarming terms hyperhidrosis and osmhidrosis. The former has been a serious annoyance in the German Army, and has, consequently, received their best attention. I have notes of two preparations largely used by the Germans with good effect. The first is a five per cent. solution of chromic acid painted over the feet once a fortnight. The second seems more directly levelled at osmhidrosis, and consists of a daily dusting with three parts salicylic acid to ninety-seven parts silicate of magnesia. Nothing, however, is to be relied on absolutely, so I will suggest a few alternative applications. In all cases begin with a thorough washing with warm water; then apply a cold solution of alum in water, or of sea salt, or of tannic acid. Or apply daily one part powdered alum to five parts glycerine; or else use one ounce of borax to two ounces of olive oil and four ounces of glycerine. One more resource may be found in the salicylic acid ointment of the British Pharmacopœia. It is evident that in all these cases the treatment is designed either to moderate the perspiration by some local astringent, or else to disinfect and deodorize it. A far better plan would be to find out the cause of the excessive secretion, and of that curious by-product of vital chemistry, which has so powerful an odor. Of several possible causes, I think that one is an impeded circulation due to tight boots. Certain it is that some persons who are ordinarily much troubled in this way, are perfectly free from any thing objectionable so long as they go barefooted. In some cases, woollen causes an odor; in others, it is the one material which can be worn without it. Here is a large assortment of plans to choose from, but the worst of it is, that one cannot guarantee that any of them will be successful.—R. A. CHUDLEIGH, *Wimborne.*

